# Regulation of mRNA Translation by MID1: A Common Mechanism of Expanded CAG Repeat RNAs

**DOI:** 10.3389/fncel.2016.00226

**Published:** 2016-10-07

**Authors:** Nadine Griesche, Judith Schilling, Stephanie Weber, Marlena Rohm, Verena Pesch, Frank Matthes, Georg Auburger, Sybille Krauss

**Affiliations:** ^1^German Center for Neurodegenerative DiseasesBonn, Germany; ^2^Experimental Neurology, Goethe University Medical SchoolFrankfurt, Germany

**Keywords:** MID1, polyglutamine diseases, CAG repeat expansion, RNA-toxicity, RNA-binding proteins

## Abstract

Expansion of CAG repeats, which code for the disease-causing polyglutamine protein, is a common feature in polyglutamine diseases. RNA-mediated mechanisms that contribute to neuropathology in polyglutamine diseases are important. RNA-toxicity describes a phenomenon by which the mutant CAG repeat RNA recruits RNA-binding proteins, thereby leading to aberrant function. For example the MID1 protein binds to mutant *huntingtin* (*HTT*) RNA, which is linked to Huntington's disease (HD), at its CAG repeat region and induces protein synthesis of mutant protein. But is this mechanism specific to HD or is it a common mechanism in CAG repeat expansion disorders? To answer this question, we have analyzed the interaction between MID1 and three other CAG repeat mRNAs, *Ataxin2* (*ATXN2*), *Ataxin3* (*ATXN3*), and *Ataxin7* (*ATXN7*), that all differ in the sequence flanking the CAG repeat. We show that *ATXN2, ATXN3*, and *ATXN7* bind to MID1 in a CAG repeat length-dependent manner. Furthermore, we show that functionally, in line with what we have previously observed for HTT, the binding of MID1 to *ATXN2, ATXN3*, and *ATXN7* mRNA induces protein synthesis in a repeat length-dependent manner. Our data suggest that regulation of protein translation by the MID1 complex is a common mechanism for CAG repeat containing mRNAs.

## Introduction

CAG repeat expansion diseases are the most common forms of inherited neurodegenerative diseases. They are caused by expansion mutations of the trinucleotide CAG in the respective disease-causing genes. These genes are functionally unrelated and the only common motif of these genes is the CAG repeat. If the CAG repeat is located within the protein-coding region it encodes a polyglutamine stretch. Intraneuronal aggregation of the polyglutamine proteins is a pathological hallmark of several CAG repeat diseases and the production of polyglutamine proteins is linked to neurotoxicity (Rudnicki and Margolis, 2003; Shao and Diamond, 2007; Fiszer and Krzyzosiak, 2014). In addition to neurotoxic effects of polyglutamine protein, there is emerging evidence showing that RNA-mediated mechanisms also contribute to neurotoxicity in polyglutamine diseases (reviewed in Nalavade et al., 2013 and Schilling et al., 2016).

Structurally, RNAs with expanded CAG repeats fold into hairpin structures *in vitro* and these hairpins increase in size and stability with increasing CAG repeat numbers (Sobczak et al., 2003; Sobczak and Krzyzosiak, 2005; Kiliszek et al., 2010; de Mezer et al., 2011). These RNA molecules with expanded CAG repeats can execute abnormal functions by recruiting different RNA binding proteins leading to the loss of normal function of these proteins and/or inducing aberrant function of these proteins when bound to the CAG repeat RNA (reviewed in Nalavade et al., 2013). For example, there is evidence that polyglutamine protein synthesis from expanded CAG repeat mRNAs is increased compared to CAG repeat mRNAs with normal repeat lengths (Krauss et al., 2013). Of note, this affects not only the polyglutamine protein, but additionally also homopolymeric expansion proteins are produced from expanded CAG repeat mRNA in all three reading frames without an AUG start codon by RAN translation (repeat-associated non-ATG translation) (Bañez-Coronel et al., 2015). Based on the neurotoxic function of all these protein species produced from expanded CAG repeats, a reduction of these proteins will be beneficial in the disease context. In accordance, reduction of polyglutamine protein in disease models for polyglutamine diseases improved the disease phenotype (Yamamoto et al., 2000; Boudreau et al., 2009; Zhang and Friedlander, 2011). But how is the increased protein synthesis rate from expanded CAG repeat mRNAs regulated?

One mechanism that we have recently identified to play a role in regulating the translation of *huntingtin* (*HTT*) mRNA with expanded CAG repeats involves the MID1-protein phosphatase 2A (PP2A) complex (Krauss et al., 2013). MID1 is an E3 ubiquitin ligase: upon binding to PP2A, MID1 catalyzes the ubiquitin-dependent degradation of PP2A (Trockenbacher et al., 2001). Thereby MID1 acts as a negative regulator of PP2A activity and at the same time unleashes the activity of the PP2A opposing kinase mTOR (Liu et al., 2011). mTOR and PP2A regulate the phospho-dependent activity of S6K, a translational regulator. Via MID1, S6K is recruited to the expanded CAG repeat motif of *HTT* mRNA. This recruitment of the MID1 complex to the expanded mutant *HTT* mRNA induces translation in a CAG repeat length-dependent manner (Krauss et al., 2013).

Here we addressed the question of whether an MID1-dependent increase in translation of expanded CAG repeat mRNA is specific to *HTT* or if this is a common feature of CAG repeat expansion disorders. mRNAs with expanded CAG repeats can fold into hairpins (Sobczak et al., 2003; Michlewski and Krzyzosiak, 2004; Kiliszek et al., 2010; de Mezer et al., 2011). However, there is a CCG repeat down stream of the CAG repeat of HTT that can stabilize this hairpin structure. A similar CCG repeat is not present in other CAG repeat mRNAs, such as *Ataxin2* (*ATXN2*), *Ataxin3* (*ATXN3*), or *Ataxin7* (*ATXN7*). In this study we show that MID1 can also bind to the CAG repeat region of *ATXN2, ATXN3*, and *ATXN7 in vitro*, suggesting that binding of MID1 to CAG repeats is not dependent on the flanking regions. Furthermore, we show that this binding of MID1 to *ATXN2, ATXN3*, and *ATXN7* mRNA, similar to what we have shown previously for HTT (Krauss et al., 2013), induces translation in a CAG repeat length-dependent manner *in vitro* and in cell lines. Our data suggest that MID1 is a common regulator of CAG repeat mRNAs and thus may be a disease modifier in CAG repeat expansion disorders. This observation makes the MID1 complex an interesting putative therapeutic target for the treatment of polyglutamine diseases.

## Methods

### RNA-protein-co-immunoprecipitation

Primary cortical neurons from a SCA3 mouse model [B6;CBA-Tg(ATXN3^*^)84.2Cce/IbezJ (JAX labs)] were prepared from embryos at E14 as described previously (Kickstein et al., 2010). These transgenic mice express human mutant ATXN3 containing 84 CAG repeats. Cells were transfected with pCMVTag2a-MID1 using Lipofectamine 2000. 48 h after the transfection cells were harvested, UV-crosslinked (300 J/cm^2^) and lysed in TKM buffer (20 mM Tris, 50 mM KCl, 5 mM MgCl_2_). MID1-containing RNA-protein complexes were purified by immunoprecipitation using ANTI-FLAG M2 Affinity Gel (SIGMA-ALDRICH) or Mouse IgG-Agarose (SIGMA-ALDRICH) as a negative control. To isolate MID1-bound RNAs the immunoprecipitates were treated with proteinase K for 30 min at 37°C and RNA extraction was performed by phenol/chloroform extraction followed by ethanol precipitation. cDNA synthesis was performed using the TaqMan Reverse transcription reagent kit (LifeTechnologies). As a negative control, reverse transcriptase was not used (−RT). PCR was performed using the following primers to amplify human *ATXN3*: forward primer 5′-TGGCTCAATTAC AACAGGAAGGT-3′ and reverse primer 5′-TGGTCG ATGCATCTGTTGGA-3′ (PCR product 113 bp). Similarly, mouse embryonic fibroblasts of a homozygous knock-in mouse model expressing mutant *ATXN2* with 42 CAG repeats (Damrath et al., 2012), were subjected to MID1-immunprecipitation followed by extraction of MID1-bound RNAs and RT-PCR analysis using primers for *ATXN2*: forward primer 5′-GCATGTCCCAAATTAC CATACAAC-3′ and reverse primer 5′-CCGGTGGAA ATGGCAAAGTAGA-3′ (PCR product 65 bp).

### RNA pulldown

*ATXN2, ATXN3*, or *ATXN7* fragments containing the CAG repeat plus flanking region with either normal or mutant repeat lengths were PCR amplified (ATXN2-forward 5′-CCAAGCTTCTAATACGACTCACTATAGGGAGACCTCACCATGTCGCTGA-3′, ATXN2-reverse 5′-TGTTACTGTTTCGACCTCTGC-3′; AT XN3-forward 5′-CCAAGCTTCTAATACGACTCACTATAGGGAGACCGCAGGGCTATTCAGCTAAG-3′, ATXN3-reverse 5′-CAGCTGCCTG AAGCATGTCTT-3′; ATXN7-forward 5′-CCAAGCTTCTAATACGACTCACTATAGGGAGAGAATGTCGGAGCGGGCCG-3′, ATXN7-reverse 5′-CCCAGCATCA CTTCAGGACT-3′). As templates plasmids containing the complete cDNA of the different ATXNs from either SCA patients or healthy individuals were used. The repeat numbers of the different cDNAs were: *ATXN2* normal (CAG)_8_-CAA-(CAG)_4_-CAA-(CAG)_8_, *ATXN2* mutant (CAG)_74_, *ATXN3* normal (CAG)_15_, *ATXN3* mutant (CAG)-CAA-(CAG)_40_-CGG-(CAG)_21_, *ATXN7* normal (CAG)_19_, *ATXN7* mutant (CAG)_43_-CGG-(CAG)_37._ All forward primers contained the T7 promotor sequence to allow subsequent *in vitro* transcription of the PCR product. Purified PCR products were *in vitro* transcribed using the RiboMAX Express large scale RNA production system-T7 (Promega) according to the manufacture's instructions with some modifications. Briefly, 2 μg of purified PCR product were transcribed for 4 h at 37°C under the addition of biotinylated UTPs (Ambion). The resulting *in vitro* transcribed RNA was purified by phenol/chloroform extraction followed by ethanol precipitation. MID1-FLAG was overexpressed in HeLa cells. Cells were lysed in TKM buffer and lysates were incubated with the *in vitro* transcribed RNA. Subsequently, the mixture was incubated with M280 streptavidin coated magnetic beads (Thermo Fisher). After extensive washing, RNA-bound proteins were eluted by boiling the beads in 80 μl sample buffer (48% urea, 15 mM Tris-HCL pH 7.5, 8.7% glycerine, 1% SDS, 0.004% Bromophenolblue, 143 mM Mercaptoethanol), for 10 min at 95°C. RNA-bound proteins were then analyzed on western blots using ANTI-FLAG M2-Peroxidase (HRP) antibody (SIGMA, A8592) to detect FLAG-MID1.

### *In vitro* translation

Firefly luciferase constructs fused to *ATXN2, ATXN3*, or *ATXN7* fragments containing the CAG repeat plus flanking region with either normal or mutant repeat lengths were PCR amplified using the following primers: Luci T7 for 5′-CCA AGCTTCTAATACGACTCACTATAGGGAGAATGGAAGACGCCAAAAACA TA-3′; ATXN2-reverse 5′-TGTTACTGTTTCGAC CTCTGC-3′; ATXN3-reverse 5′-CAGCTGCCTGAA GCATGTCTT-3′; ATXN7-reverse 5′-CCCAGC ATCACTTCAGGACT-3′. As templates Plasmids with the different ATXN fragments described in the section for luciferase assays were used. All forward primers contained the T7 promotor sequence to allow subsequent *in vitro* transcription of the PCR product. The PCR products were *in vitro* transcribed using the T7 RiboMAX™ Express Large Scale RNA Production System (Promega). After phenol/chloroform extraction and subsequent ethanol precipitation of the resulting RNA an *in vitro* translation reaction, using the Flexi Rabbit Reticulocyte Lysate System (Promega), was performed. The amount of *in vitro* translated firefly luciferase was measured in a luciferase assay using the protocol described below without the Stop and Glow reaction. As a positive control we included previously published constructs (Krauss et al., 2013) with *HTT* exon1 fused to luciferase, and as a negative control we used a sample without RNA.

### Luciferase assays

For the Luciferase assays HEK293T cells were used. Cells were cultivated in DMEM medium (Gibco) containing 10% FCS (PAN) and Pen/Strep. Cells were tested semi-annually for mycoplasma using the Mykoplasma Kit from Promokine (PK-CA91-1096). Cells were transfected with psiCheck2-constructs in which short *ATXN2, ATXN3*, and *ATXN7* sequences containing either normal or mutant CAG repeats were cloned into the 3′-UTR of firefly luciferase. This allows the analysis of effects mediated by CAG repeats in the mRNA without being translated into polyglutamine stretches fused to the luciferase. This is important since polyglutamine stretches might make the luciferase insoluble and might therefore influence its activity. On the same vector backbone there is a renilla luciferase gene, which is used as internal transfection control. Additionally, we created constructs in which pure CAG or CAA repeats (50 repeats each), were fused to renilla luciferase in the psiCheck2 vector. In these plasmids firefly luciferase served as an internal transfection control. 24 h after transfection cells were lysed in passive lysis buffer (Promega). For the luciferase measurements the Dual luciferase assay kit (Promega) was used. In brief, in a first step a substrate for firefly luciferase (LARII) was added to the lysates and the relative light units were measured. After the measurement of the firefly luciferase, an equal amount of renilla substrate (Stop and Glo buffer) was added to the samples. After a 2 s delay, renilla luciferase was measured. Mean values of relative light units ± SEM were calculated. The average of the *ATXN2, ATXN3*, and *ATXN7* samples with normal CAG repeats was set to 100%. Statistics were done using *t*-test.

### MID1 knock down

1 × 10^5^ HEK293T cells per well of a 24 well plate were seeded 1 day prior transfection. Cells were transfected with 2.5 μl of a 20 μM stock of either a pool of four siRNAs targeting MID1 (TTGAGTGAGCGCTATGACAAA, AAGGTGATGAGGCTTCGCAAA, CACCGCAUCCUAGUAUCACACTT, CAGGAUUAC AACUUUUAGGAATT) or a non-silencing control siRNA (AATTCTCCGAACGTGTCA CGT) per well using Oligofectamine (ThermoFisher) according to the manufacturer's instructions. After 24 h cells were transfected with the psiCheck2 constructs described above with Lipofectamine 2000 (ThermoFisher) according to the manufacturer's instructions. After another 24 h cells were harvested in passive lysis buffer (Promega). Dual luciferase assays were performed as described above. Average values of the relative light units normalized to the internal transfection control are shown. The resulting values were normalized to the relative light units of control samples, in which empty psiCheck2 vector was co-transfected with the respective siRNAs. Statistics were done using *t*-test.

### Fluorescence recovery after photobleaching (FRAP) based assay to monitor translation

To monitor the translation rate of the different CAG repeat mRNAs with a previously established FRAP based technique in living cells (Krauss et al., 2013), we cloned the sequence of enhanced green fluorescent protein (EGFP) into the pBUD-CE4 vector backbone. 3′ of the EGFP sequence behind the stop codon in the untranslated region, we cloned the short *ATXN2* or *3* sequences containing either normal or mutant CAG repeats described in Supplementary Table 1. For the FRAP-based assays HELA cells were used. Cells were cultivated in DMEM medium (Gibco) containing 10% FCS (PAN) and Pen/Strep. Cells were tested semi-annually for mycoplasma using the Mykoplasma Kit from Promokine (PK-CA91-1096). 1 × 10^3^ HeLa cells per well were grown on microscopy 8-well chamber slides and were transfected with these constructs using Polyfect (Qiagen) according to the manufacturer's instructions. 20 h after transfection cells were analyzed with an LSM700 microscope from Zeiss using a X20 objective. Individual cells were chosen and the GFP signal was bleached with a high intensity 488 argon laser. After bleaching the GFP signal was imaged every 5 min for 4 h. For the analysis the GFP signal of every cell was analyzed as the sum of the pixel over the area of the cell. To normalize the results of each cell, the second frame after bleaching was set as point zero and put to 100%.

### Western blot

Cell pellets of a fibroblast cell line of a previously described SCA3 patient (MJD1) with 74/22 CAG repeats in the *ATXN3* gene (Koch et al., 2011) were analyzed. Protein extracts were dissolved sample buffer (48% urea, 15 mM Tris-HCL pH 7.5, 8.7% glycerine, 1% SDS, 0.004% Bromophenolblue, 143 mM Mercaptoethanol), boiled for 20 min at 95°C, separated on 4–20% Mini-PROTEAN TGX Precast Protein Gels (Bio-Rad) and western blotted onto PVDF membranes (Roche). ATXN3 was detected using anti-ATXN3 antibodies (Millipore, MAB5360) and actin (Cell signaling, 4967L) was detected as loading control. Bands on the western blots were analyzed in triplicates and quantified using Fiji Software. Data shown represent mean ± SEM. Statistical significances were evaluated using *t*-test (twotailed, homoscedastic).

### Prediction of RNA secondary structures

Prediction of RNA secondary structure was performed using the mfold Web Server (http://unafold.rna.albany.edu/?q=mfold) (Mathews et al., 1999; Zuker, 2003). To determine secondary RNA structures the parameters were set to assume a 1M NaCl solution, 37°C and a linear sequence. Free energy contributions of the folding were counted and are listed.

## Results

### MID1 binds to several CAG repeat RNAs

**Figure 1:**
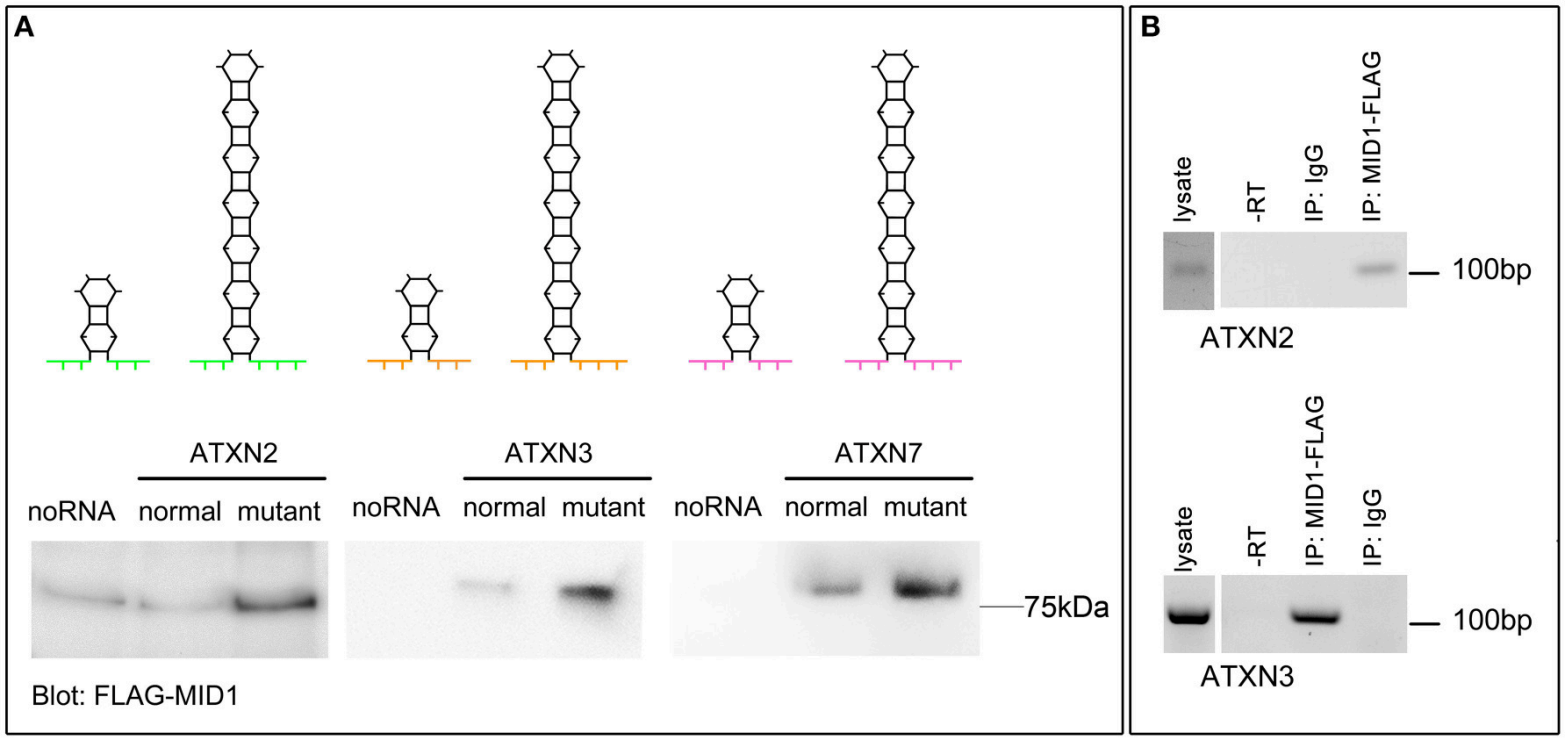
**MID1 binds to CAG repeat RNAs irrespective of the repeat-flanking sequences. (A)** The binding of three different CAG repeat RNAs to MID1 was analyzed. Upper panel: schematic showing the predicted hairpin folding as well as the different CAG repeat flanking regions of ATXN2, ATXN3, and ATXN7 to visualize the different RNA fragments that were used, different colors symbolize the different flanking regions. Lower panel: *in vitro* transcribed fragments of *ATXN2, ATXN3*, and *ATXN7* containing either normal or mutant CAG repeats were incubated with MID1-FLAG containing lysates. RNA-bound MID1 protein was analyzed on western blot using FLAG-antibodies. A representative western blot of *n* = 3 experiments is shown. **(B)** RNA-immunoprecipitation was performed in lysates from either mouse embryonic fibroblasts expressing mutant *ATXN2* or primary neurons of a SCA3 mouse model that expresses full-length human *ATXN3* with a mutant CAG repeat. MID1 was immunopurified and the presence of *ATXN2* (upper panel) or human *ATXN3* (lower panel) in the MID1 complex was tested by RT-PCR on RNA that co-purified with MID1. As negative control, a sample with unspecific IgG was used. As additional negative control a–RT reaction was performed. RT-PCR products were analyzed on an agarose gel. A representative gel of *n* = 3 experiments is shown.

MID1 binds to *HTT* mRNA at its CAG repeat region and induces its translation (Krauss et al., 2013). To test if the flanking regions influence the binding of MID1 to CAG repeat mRNAs, we tested the binding of MID1 to *ATXN2, ATXN3*, and *ATXN7* mRNAs. We performed RNA-protein binding assays, in which *in vitro* transcribed *ATXN2, ATXN3*, and *ATXN7* RNA-constructs with either normal or mutant CAG repeat lengths were used. For a detailed description of the RNA sequences and the predicted RNA folding see Supplementary Figure 1 and Supplementary Table 1. These *in vitro* transcribed RNAs were immobilized on magnetic beads and incubated with protein extracts containing FLAG-MID1 to allow RNA-protein binding. After extensive washing, MID1 that bound to respective RNA was analyzed on a western blot using FLAG-antibodies. In all three cases, we observed that (i) MID1 did bind to the CAG repeat RNA and (ii) this binding was stronger in the samples with expanded CAG repeats (Figure 1A). This experiment suggests that the MID1 protein complex binds to CAG repeat RNAs independent of the flanking regions. To further establish this and to test for the functional relevance of this with an RNA that is naturally processed (e.g., transcribed, spliced, etc.) and that is linked to a polyglutamine disease, we performed experiments on ATXN3 using neurons from a SCA3 mouse model and experiments on ATXN2 using mouse embryonic fibroblasts of a SCA2 mouse model. To test if MID1 binds to endogenously transcribed full-length mutant *ATXN2* mRNA, we expressed FLAG-MID1 in mouse embryonic fibroblasts of a homozygous knock-in mouse model expressing mutant *ATXN2* with 42 CAG repeats (Damrath et al., 2012). From these cells MID1-protein complexes were immunopurified and MID1 bound RNAs were isolated. The presence of *ATXN2* mRNA in the MID1-immunoprecipitates were then analyzed by RT-PCR. Similarly, to test if MID1 binds to endogenously transcribed full-length mutant *ATXN3* mRNA, we expressed FLAG-MID1 in primary neurons of a transgenic mouse line that expresses full-length mutant human *ATXN3*. From these cells MID1-protein complexes were immunopurified and MID1 bound RNAs were isolated. The presence of mutant human *ATXN3* mRNA in the MID1-immunoprecipitates were then analyzed by RT-PCR using primers specific for human ATXN3. In line with the data from the RNA-protein pulldown, we clearly observed binding between MID1 and full-length mutant *ATXN3* mRNA as well as full-length *ATXN2* mRNA (Figure 1B).

### Translation of mutant *ATXN2, ATXN3*, and *ATXN7* is increased

**Figure 2:**
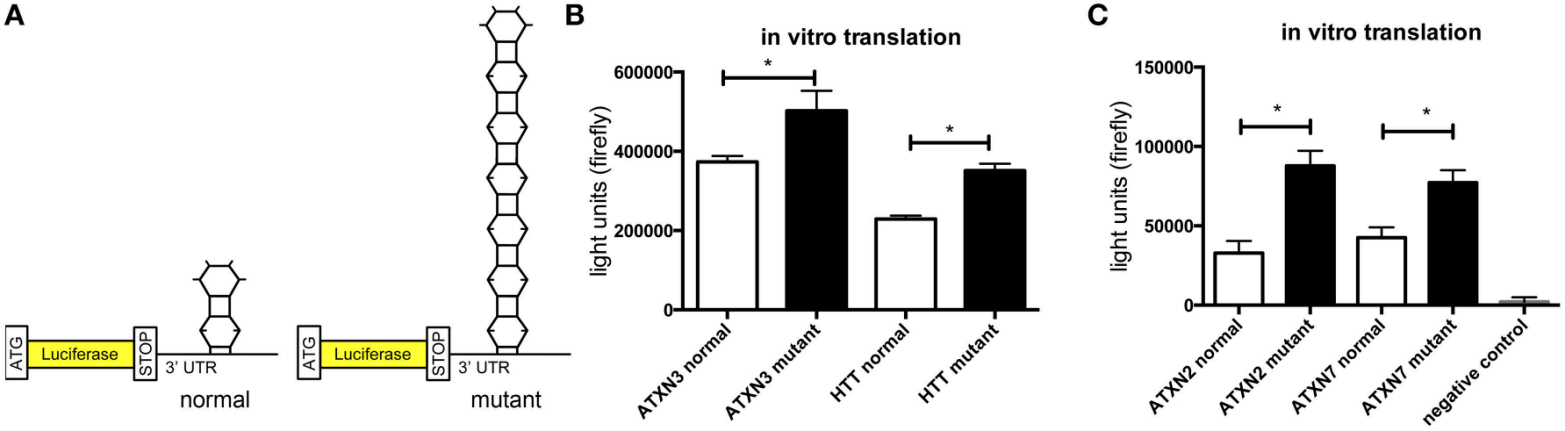
**Increased translation of *ATXN2*, *ATXN3*, and *ATXN7* constructs with expanded CAG repeats *in vitro*. (A)** Schematic showing luciferase reporter constructs used in this experiment. Luciferase reporter constructs were cloned that contain the CAG repeat region of *ATXN2, ATXN3*, and *ATXN7* in the 3′UTR. Two constructs each with either normal (left) or mutant CAG (right) repeat lengths were cloned. **(B)** *In vitro* translation of the luciferase reporters of *ATXN3*. Previously published *HTT* exon1 luciferase reporter constructs were included as positive controls (Krauss et al., 2013). The constructs with either normal or mutant CAG repeat lengths were *in vitro* transcribed and equimolar amounts of RNA were subjected to *in vitro* translation. The level of translated luciferase reporter was measured in a luciferase assay. Columns represent mean values ± SEM. *n* = 3, *p* ≤ 0.01. **(C)** *In vitro* translation of the luciferase reporters of *ATXN2* and *ATXN7*. The constructs with either normal or mutant CAG repeat lengths were *in vitro* transcribed and equimolar amounts of RNA were subjected to *in vitro* translation. The level of translated luciferase reporter was measured in a luciferase assay. As a negative control, a sample without RNA was included. Columns represent mean values ± SEM. *n* = 3, *p* ≤ 0.0001. ^*^Indicates statistically significant differences (*p* < 0.05).

**Figure 3:**
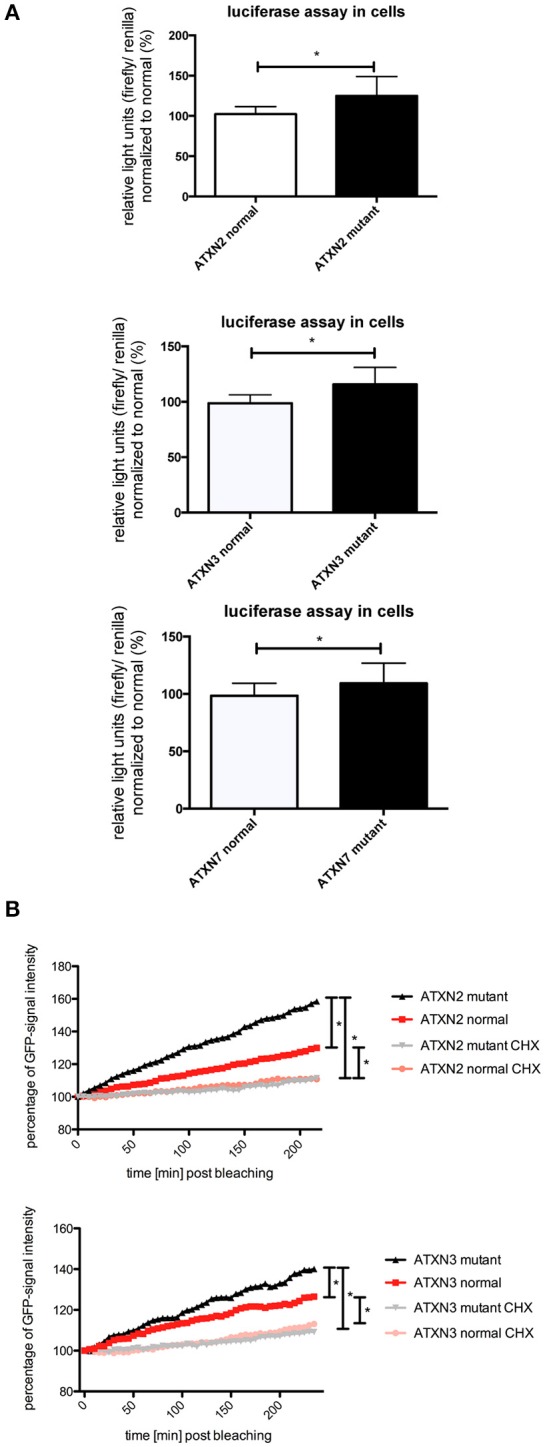
**Enhanced translation of *ATXN2*, *ATXN3*, and *ATXN7* constructs with expanded CAG repeats in cell lines. (A)** Dual luciferase reporter assay in HEK293T cells transfected with constructs containing a firefly luciferase reporter with the CAG repeat region of *ATXN2, ATXN3*, and *ATXN7* in the 3′UTR and renilla luciferase as an internal transfection control. Data shown represent relative light units of the *ATXN2, ATXN3*, and *ATXN7* firefly luciferase construct normalized to renilla luciferase. The mean of the constructs with normal CAG repeat length was set to 100%. Columns represent mean values ± SEM. *n* = 6, *p* < 0.05 **(B)** Fluorescence recovery after photobleaching assays to monitor translation were performed for *ATXN2* and *ATXN3* containing normal or mutant CAG repeat regions. HeLa cells transfected with GFP-ATXN2 normal or mutant as well as GFP-ATXN3 normal or mutant were bleached with a high intensity laser. Translation is monitored by analyzing the recovery of GFP signal in living cells over 4 h. Negative controls are cells treated with the translational inhibitor cycloheximide. Lines represent mean values over the time. Assays were performed in triplicates with several cells per experiments (cells analyzed altogether —*ATXN2*: n_mutant_ = 17, n_normal_ = 12, n_mutant CHX_ = 11, n_normal CHX_ = 19; *ATXN3*: n_mutant_ = 21, n_normal_ = 20, n_mutant CHX_ = 16, n_normal CHX_ = 18). Statistics were performed using two way anova (*p* < 0.0001). ^*^Indicates statistically significant differences (*p* < 0.05).

In previous studies we observed that the translation rate of *HTT* increases with increasing CAG repeat length (Krauss et al., 2013). Therefore, we asked if similarly the translation of *ATXN2, ATXN3*, and *ATXN7* with mutant compared to normal CAG repeat length is increased. To test this, we performed an *in vitro* translation assay. For this, luciferase reporter constructs containing *ATXN2, ATXN3*, and *ATXN7* with either normal or mutant CAG repeat lengths in the 3′ UTR were *in vitro* transcribed. The position of the *ATXN* fragments in the 3′UTR was chosen to allow detection of regulatory effects of the CAG repeats on the RNA level without being translated into polyglutamine stretches on the protein level (Figure 2A). Equimolar amounts of the resulting *in vitro* transcribed RNAs were then used in an *in vitro* translation reaction. The level of luciferase reporter that was translated was measured in a luciferase assay. As a positive control we included previously published constructs (Krauss et al., 2013) with HTT exon1 fused to luciferase, and as a negative control we used a sample without RNA. The translation rate of the reporter-containing mutant *ATXN2, ATXN3*, and *ATXN7* was significantly increased compared to the constructs with normal repeat length (Figure 2B). In a second set of experiments, we used constructs containing a firefly luciferase reporter with the CAG repeat region of *ATXN2, ATXN3*, and *ATXN7* in the 3′UTR and renilla luciferase as an internal transfection control to transfect HEK293T cells and measure the protein level produced in living cells. In line with our observation from the *in vitro* translation, the level of the luciferase translated from the *ATXN2, ATXN3*, and *ATXN7* mutant RNA was significantly increased compared to the normal control (Figure 3A). Additionally we performed a previously established FRAP-based assay that allows monitoring of translation in living cells (Krauss et al., 2013). In brief, GFP-constructs containing *ATXN2* or *ATXN3* with either normal or mutant CAG repeat lengths in the 3′ UTR were transfected into HeLa cells. After bleaching of the GFP protein in the entire cell, we measured GFP-signal recovery, which corresponds to newly synthesized protein over a time frame of 4 h. To control that the GFP-signal recovery indeed represents freshly translated protein, we performed experiments with the translation inhibitor cycloheximide (CHX). In line with the data from the luciferase assay, we observed a significantly increased translation rate of mutant compared to normal *ATXN2* and *ATXN3* (Figure 3B, Supplementary Figure 2). Together these data suggest that, similar to what we have seen for *HTT* (Krauss et al., 2013), the translation rate of mutant *ATXN2, ATXN3*, and *ATXN7* is increased.

### MID1 regulates translation of CAG repeat mRNAs

**Figure 4:**
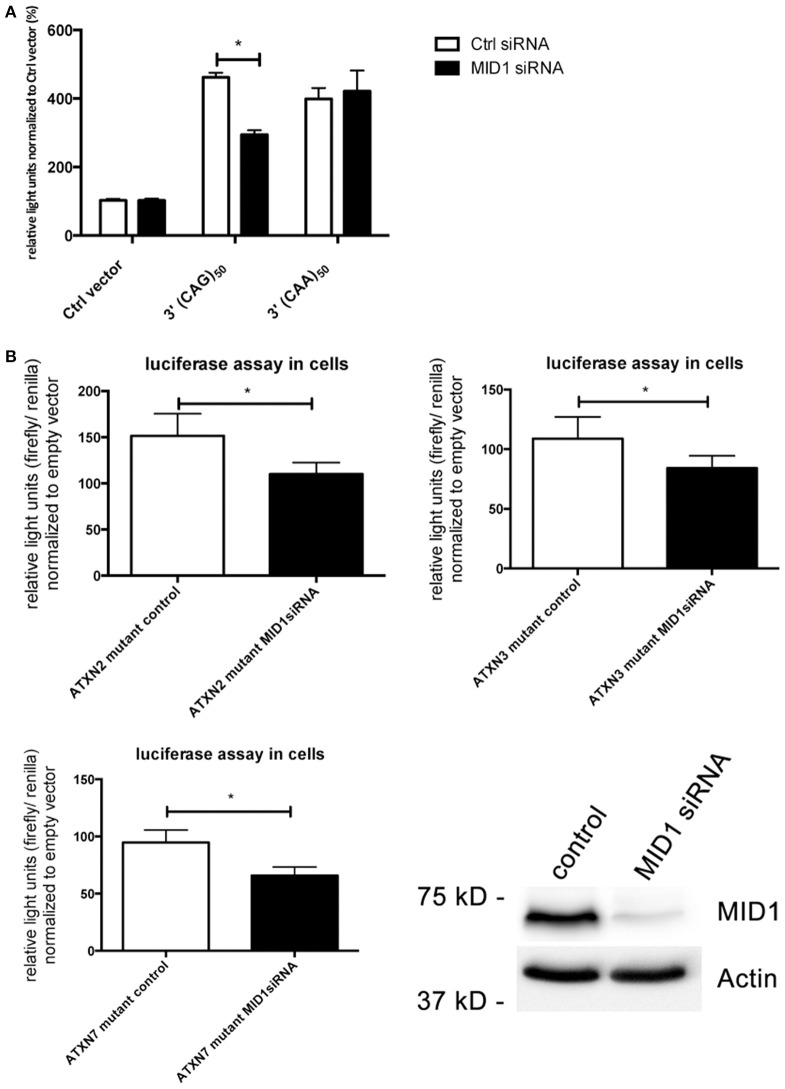
**Translation of CAG repeats but not CAA repeats is regulated by MID1. (A)** Dual luciferase reporter assay in HEK293T cells co-transfected with MID1 specific or control siRNAs and constructs containing a renilla luciferase reporter with pure CAG repeats or pure CAA repeats in the 3′UTR and firefly luciferase as an internal transfection control. Data shown represent relative light units of renilla luciferase construct normalized to firefly luciferase. Columns represent mean values ± SEM. *n* = 3, *p* < 0.0001. **(B)** Dual luciferase reporter assay in HEK293T cells co-transfected with MID1 specific or control siRNAs and constructs containing a firefly luciferase reporter with the CAG repeat region of *ATXN2, ATXN3*, and *ATXN7* in the 3′UTR and renilla luciferase as an internal transfection control. Data shown represent relative light units of the *ATXN2, ATXN3*, and *ATXN7* firefly luciferase construct normalized to renilla luciferase. Values were normalized to mean values of empty vectors co-transfecetd with the respective siRNA. Columns represent mean values ± SEM. *n* = 6, *p* < 0.0001. To show knockdown efficiency MID1 (upper panel) and actin (lower panel, loading control) were detected on a western blot using specific antibodies. ^*^Indicates statistically significant differences (*p* < 0.05).

In previous studies we observed that binding of the MID1-complex to mutant *HTT* mRNA leads to an increased translation (Krauss et al., 2013). Our finding that also the translation rate of mutant *ATXN2, ATXN3*, and *ATXN7* is increased led us to the hypothesis that MID1 regulates and induces translation of CAG repeat mRNAs irrespective of the repeat flanking sequence. To test this hypothesis, we created luciferase constructs, which are fused to pure CAG repeats in their 3′UTR. As a control we used constructs with CAA repeats of similar length. These constructs were transfected into HEK293T cells that underwent siRNA mediated MID1-knockdown. To minimize off-target effects we used a pool of four different siRNAs. Strikingly, MID1 depletion reduced the translation of the construct with the pure CAG repeat, while it did not influence the construct with the CAA repeat (Figure 4A). To show if similar to the reporter with pure CAG repeats, the increased translation of mutant *ATXN2, ATXN3*, and *ATXN7* is MID1 dependent we co-transfected the above-mentioned luciferase reporters fused to mutant *ATXN2, ATXN3*, and *ATXN7* with or without MID1-specific siRNAs. Indeed, depletion of MID1 led to a significant reduction in the translation rate of the mutant *ATXN2, ATXN3*, and *ATXN7* reporters (Figure 4B). Of note, while the translation of the luciferase reporter with normal ATXN2 was not affected by MID1 knockdown, both luciferase reporters with normal ATXN3 and ATXN7 showed also decreased signals after MID1 depletion (data not shown). Together, all these observations suggest that MID1 binds to and regulates the translation of CAG repeat mRNAs irrespective of the repeat flanking regions.

**Figure 5:**
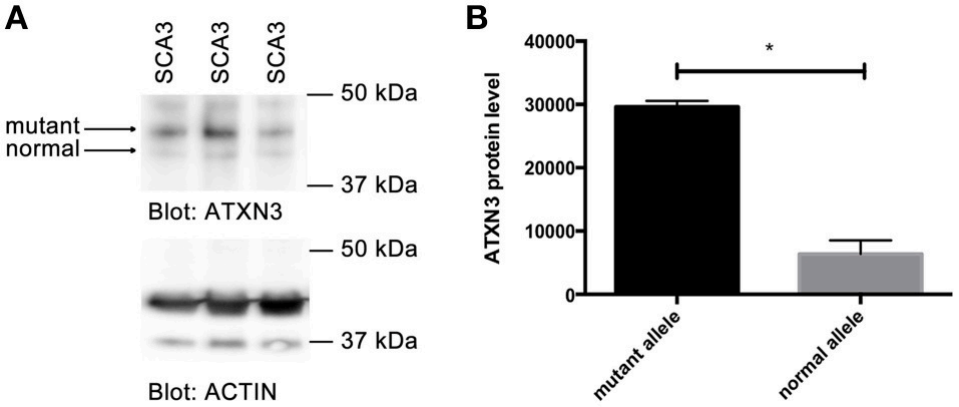
**Increased translation of mutant *ATXN3* in SCA3 fibroblasts. (A)** Western blot analysis of extracts from fibroblasts derived from a SCA3 patient carrying 74/22 CAG repeats in the *ATXN3* gene is shown. Two ATXN3 bands were detected, one smaller translated from the normal, one bigger translated from the mutant allele. Actin was analyzed as control. **(B)** Quantifications of bands (protein expressed from the normal compared with protein expressed from the mutant allele) from **(A)** are shown. Mean values ± SEM are shown, *n* = 3, *p* < 0.0001. ^*^Indicates statistically significant differences (*p* < 0.05).

Since we observed stronger binding of MID1 to CAG repeat constructs with expanded CAG repeats and an increased translation rate of constructs with normal vs. mutant CAG repeats, we asked if in patients with polyglutamine diseases more protein is being produced from the mutant compared to the normal allele. As an example for a polyglutamine disease, we analyzed a patient derived cell line of a SCA3 patient. We performed western blots analysis from a fibroblast cell line of a SCA3 patient detecting ATXN3. A smaller band corresponding to protein translated from the normal allele and a bigger band corresponding to mutant ATXN3 protein, were detected. Significantly more mutant than normal protein was detected suggesting that the translation rate of RNA from the mutant allele is higher than from the normal allele in SCA3 patients (Figure 5).

## Discussion

**Figure 6:**
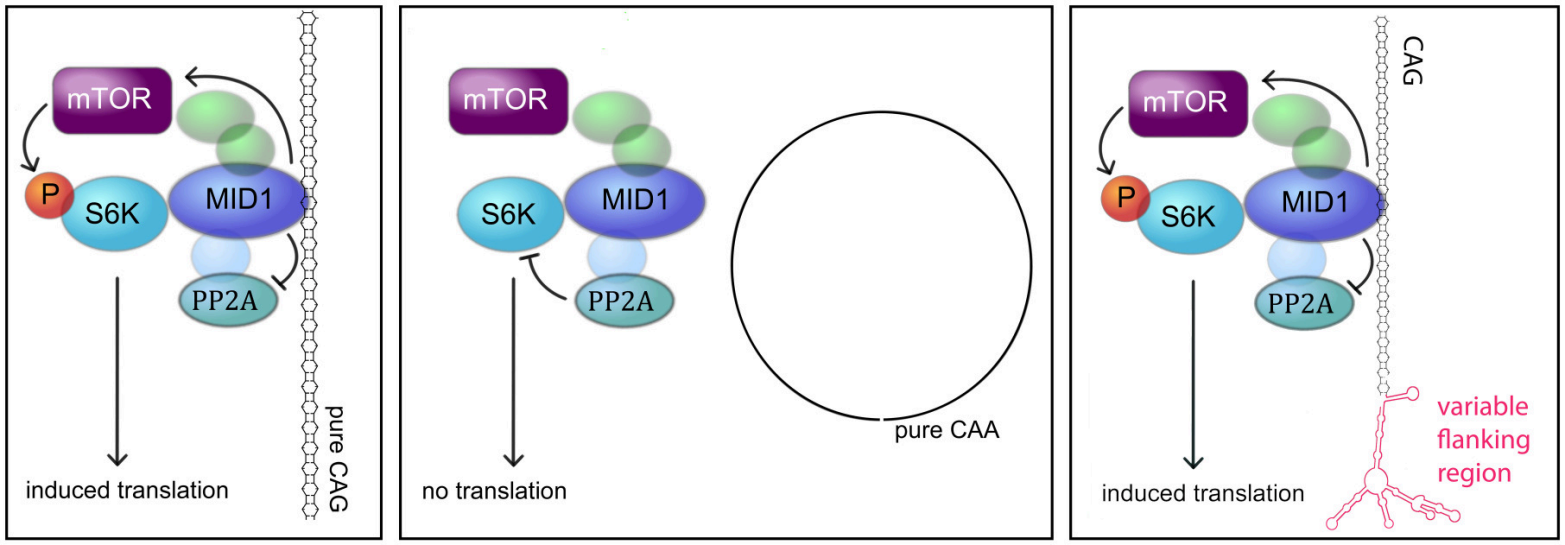
**MODEL**. Model showing the interaction between the MID1 complex and CAG repeat mRNAs (adapted from Nalavade et al., 2013). The MID1 complex binds to CAG repeats independent of its flanking region and recruits its binding partners, including S6K to the RNA. S6K is a translational regulator with phospho-dependent activity that is regulated by PP2A and mTOR. MID1 not only acts as a scaffold bridging the binding between mRNA and S6K, but it also regulates the activity of PP2A and mTOR. Thereby, MID1 is able to induce translation of mRNA.

Different molecular mechanisms have been discovered to play a role in polyglutamine diseases including RNA-mediated mechanisms. One of these mechanisms is the recruitment of the MID1 protein complex to expanded CAG repeats in the *HTT* mRNA (Krauss et al., 2013). MID1 binds to *HTT* mRNA with pathologically expanded CAG repeats and recruits a translational machinery, thereby inducing translation of mutant *HTT* mRNA (Krauss et al., 2013). In this study, we asked if this binding and translational regulation by MID1 is specific for *HTT* or if this is a common mechanism involved in several CAG repeat diseases. In summary, all our data suggest that MID1 binds to diverse CAG repeat mRNAs, independent of the flanking regions, and that this binding is accompanied by translational induction (Figure 6).

CAG repeat RNA folds into hairpin structures (Sobczak et al., 2003; Busan and Weeks, 2013). These hairpins fold due to Watson-Crick base-pairing at the G-C and C-G base pairs of the CAG sequence, interrupted by an A-A wobble (Sobczak et al., 2003; Kiliszek et al., 2010). The sequence adjacent to the CAG repeat, the flanking region, influences the folding of the hairpin structure. For example, in the *HTT* mRNA the CAG repeat borders on a CCG repeat that interacts with the CAG repeat and stabilizes the hairpin structure (de Mezer et al., 2011). In contrast, the *ATXN2, ATXN3*, and *ATXN7*mRNAs do not contain such a hairpin-stabilizing CCG motif and these CAG repeats fold into slippery hairpins, i.e., hairpins of different length exist (Michlewski and Krzyzosiak, 2004; Sobczak and Krzyzosiak, 2005). The CAG repeat hairpin structures are known to interact with certain proteins, whereupon more protein is bound by expanded CAG repeats (McLaughlin et al., 1996). This recruitment of protein to the mutant CAG repeat mRNA can lead to a loss of normal function or an abnormal function of the respective RNA-binding protein. For example muscleblind-like 1 protein (MBNL1), which normally regulates splicing of its target mRNAs, is recruited to elongated CAG repeats leading to misregulation of splicing (Yuan et al., 2007; Mykowska et al., 2011). Other examples are sequestration of nucleolin, leading to reduced cellular rRNA (Tsoi et al., 2012; Tsoi and Chan, 2013) and sequestration of proteins of the siRNA machinery, resulting in the production of short silencing RNAs that affect gene expression (Krol et al., 2007; Bañez-Coronel et al., 2012). Another example that we have shown before is the MID1 protein. Amongst other functions MID1 regulates translation of its target mRNAs (Aranda-Orgillés et al., 2011; Hettich et al., 2014; Köhler et al., 2014). MID1 is recruited to the CAG repeats of mutant *HTT* mRNA in a repeat length-dependent manner, which leads to increased translation of mutant HTT (Krauss et al., 2013). In this study we asked the question if the flanking regions of the CAG repeat, and their impact on the folding of the hairpin structure, influence the binding of MID1 to CAG repeat mRNAs. We show here that MID1 binds to elongated CAG repeats flanked by sequences of *ATXN2, ATXN3*, and *ATXN7*. This leads us to the conclusion that MID1 is recruited to mutant CAG repeat mRNAs independent of the flanking regions.

MID1 regulates translation of a number of target mRNAs (Aranda-Orgillés et al., 2011; Hettich et al., 2014; Köhler et al., 2014). For example, MID1 binds to BACE1 mRNA and induces its translation linking translational regulation by MID1 to Alzheimer's Disease (Hettich et al., 2014). Other targets of MID1-regulated translation are the PDPK-1 and androgen receptor (AR) mRNAs (Aranda-Orgillés et al., 2011; Köhler et al., 2014). Furthermore, the binding of the MID1 complex to mutant *HTT* mRNA leads to an initiation of translation via the mTOR pathway (Krauss et al., 2013). Based on this observation we asked if binding of MID1 to other CAG repeat mRNAs also increases translation. We show in this study, for the first time, evidence that *ATXN2, ATXN3*, and *ATXN7* are novel targets of MID1. Our data suggest that translation of mutant *ATXN2, ATXN3*, and *ATXN7* is similar to what we have previously seen for *HTT*, upregulated by MID1. MID1 binds to CAG repeat mRNAs in a length dependent manner. Interestingly, binding is weak to transcripts with CAG repeats of normal length, as they occur in healthy individuals. However, we observed a reduced translation of constructs with normal *ATXN3* and *ATXN7* after MID1 depletion. Therefore, it is possible that one physiological function of MID1 is the regulation of a moderate translation of CAG repeat mRNAs. However, in diseases where the CAG repeat is expanded, the binding of MID1 is increased, thus leading to an aberrantly increased translation rate.

In polyglutamine diseases several pathogenic mechanisms seem to exist in parallel and to act in concert causing neurotoxicity in a specific subgroup of neurons in patient brains. These include protein—and RNA-based mechanisms (reviewed in Nalavade et al., 2013). In a *Drosophila* model of SCA3 the interspersal of CAA within the CAG repeat (both codons code for glutamine) results in mitigated toxicity (Li et al., 2008). This observation shows that the polyglutamine protein is neurotoxic, but neurotoxicity is increased if a CAG repeat hairpin is present on the RNA level. Our data show that MID1 binds to the CAG repeats above a certain threshold, presumably the threshold where folding into hairpin structures occurs. Since MID1 induces the translation of neurotoxic polyglutamine protein this could be one explanation why pure CAG repeat mRNAs seem to be more toxic than mixed CAG-CAA mRNAs.

Another mechanism by which expanded CAG repeat mRNAs are translated at an increased rate is the so-called repeat-associated non-ATG (RAN) translation. By this mechanism not only is the polyglutamine protein translated from the CAG repeat mRNA starting at the regular AUG initiation codon, but also additional protein species that are translated directly starting at the CAG repeat without the need of an AUG start codon are produced (Zu et al., 2011; Bañez-Coronel et al., 2015). An additional mechanism that leads to the translation of polyalanine and polyserine protein species from CAG repeat mRNAs is frameshifting (Gaspar et al., 2000; Toulouse et al., 2005; Davies and Rubinsztein, 2006; Stochmanski et al., 2012). We have observed that MID1 induces translation of polyglutamine protein in a CAG repeat-length dependent manner. Future studies will show if MID1 also induces translation of protein species from the other frames like polyalanine or polyserine proteins.

To date, no effective drug that either prevents or slows progression of the polyglutamine diseases is available and current treatments are of palliative nature only. An ideal therapeutic approach should aim at reducing the level of the disease-causing polyglutamine protein. We have identified the MID1-protein as a regulator of translation of several polyglutamine proteins. Our data suggest that targeting MID1 to inhibit translation of pathogenic polyglutamine proteins could be a therapeutic approach. However, additional studies in cell line models as well as preclinical trials are needed to confirm MID1 as a putative drug target for polyglutamine diseases.

## Author contributions

NG, JS, MR, SW, VP, FM, and SK. performed experiments and analyzed the data, NG and SK. planned and designed experiments, NG and SK. wrote the paper. GA provided experimental tools. All authors discussed the results and commented on the manuscript.

### Conflict of interest statement

The authors declare that the research was conducted in the absence of any commercial or financial relationships that could be construed as a potential conflict of interest.

## Supplementary material

Supplementary Figure 1 ***In silico* prediction of secondary RNA structures**. The folding of *ATXN2, ATXN3* and *ATXN7* RNA-constructs used in this study into secondary structures was predicted using mfold software. **(A)**, **(C)**, and **(E)** prediction of *ATXN2, ATXN3* and *ATXN7* RNA secondary structures with normal CAG repeat lengths. **(B)**, **(D)**, and **(F)** prediction of *ATXN2, ATXN3* and *ATXN7* RNA secondary structures with mutant CAG repeat length. The CAG repeat region, which folds into a hairpin structure is labeled in red and repeat numbers are given.

Supplementary Figure 2 **Pictures of an exemplary FRAP experiment**. An example of a cell in the FRAP based translation assay is shown. HeLa cells were transfected with GFP-*ATXN2* with mutant CAG repeats. The seven pictures show different time points from pre-bleach up to 235 min after bleaching. The increase in GFP intensity after the bleaching (*t* = 0) reflects the translation of new protein.

Supplementary Table 1 ***ATXN2, ATXN3*, and *ATXN7*-constructs**. The sequences of the fragments of *ATXN2, ATXN3*, and *ATXN7* normal and mutant that were cloned into the reporter plasmids and were used in this study are shown.
