# Supplementary figures and images for: Regulation of mRNA Translation by MID1: A Common Mechanism of Expanded CAG Repeat RNAs
Source: Front Cell Neurosci. 2016 Oct 7;10:226. doi: 10.3389/fncel.2016.00226 (PMC5054010; doi:10.3389/fncel.2016.00226)

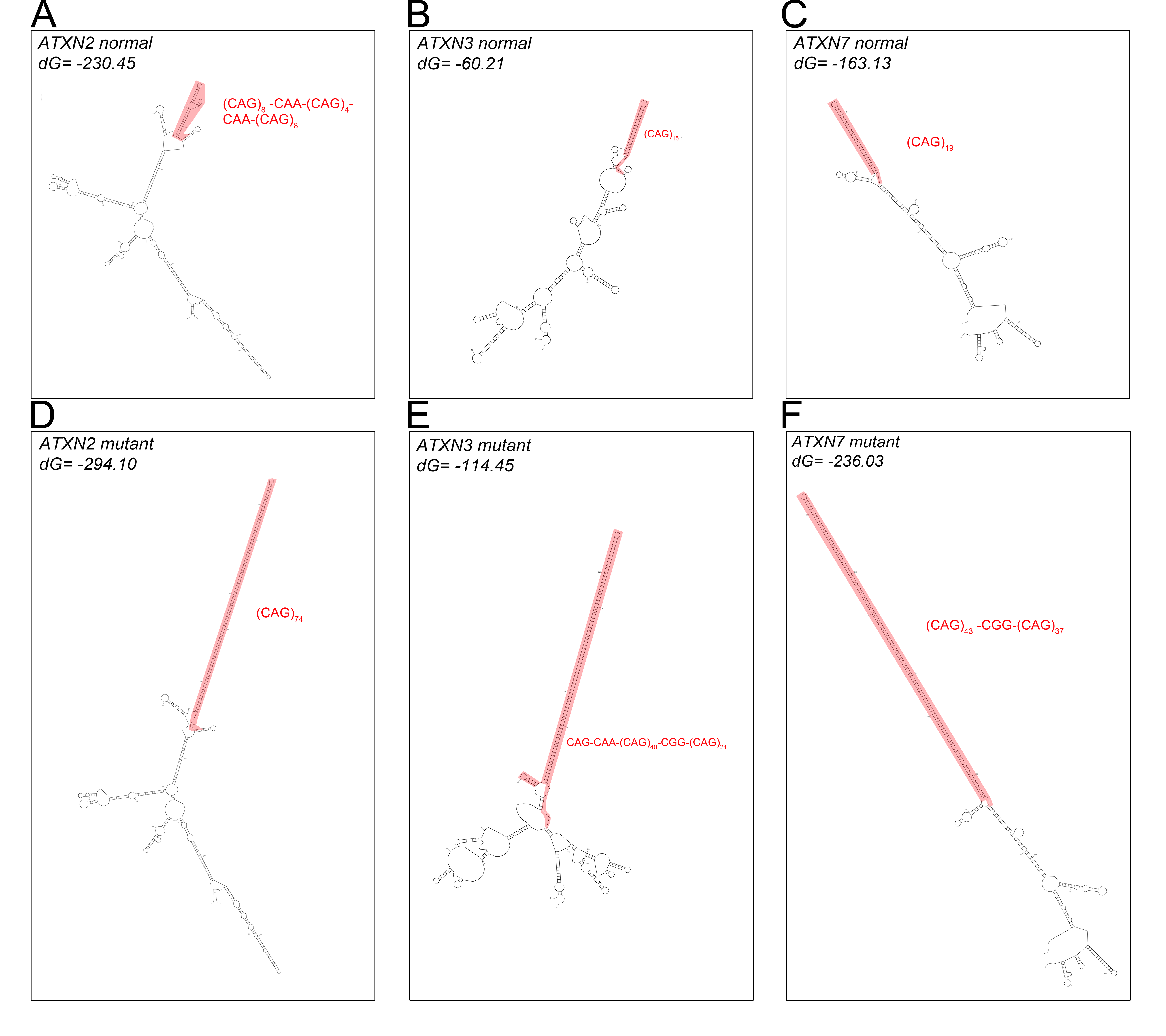

Supplement: Supplementary Figure 1 — In silico prediction of secondary RNA structures. The folding of ATXN2, ATXN3 and ATXN7 RNA-constructs used in this study into secondary structures was predicted using mfold software. (A), (C), and (E) prediction of ATXN2, ATXN3 and ATXN7 RNA secondary structures with normal CAG repeat lengths. (B), (D), and (F) prediction of ATXN2, ATXN3 and ATXN7 RNA secondary structures with mutant CAG repeat length. The CAG repeat region, which folds into a hairpin structure is labeled in red and repeat numbers are given. [file Image1.TIF]

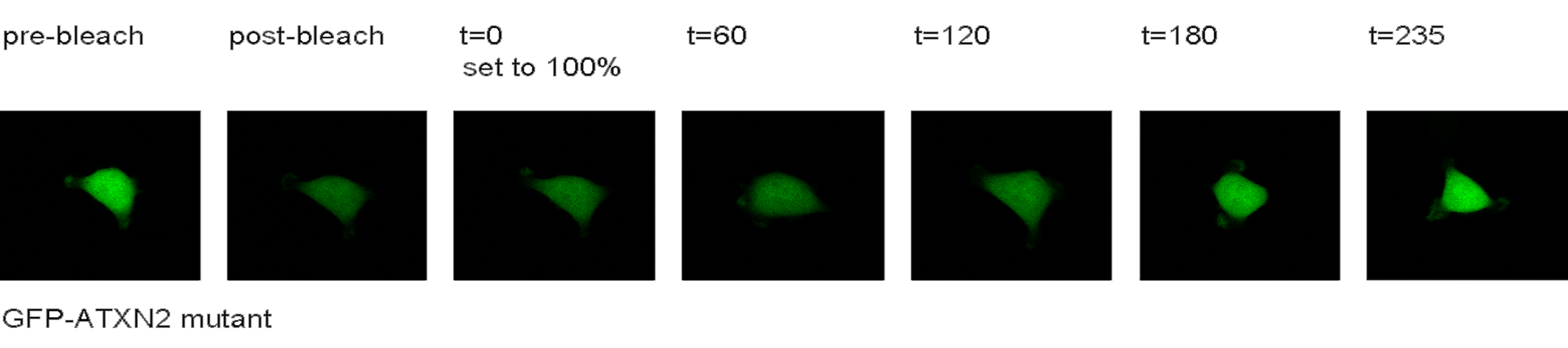

Supplement: Supplementary Figure 2 — Pictures of an exemplary FRAP experiment. An example of a cell in the FRAP based translation assay is shown. HeLa cells were transfected with GFP-ATXN2 with mutant CAG repeats. The seven pictures show different time points from pre-bleach up to 235 min after bleaching. The increase in GFP intensity after the bleaching (t = 0) reflects the translation of new protein. [file Image2.TIF]
