# Supplementary material for: Regulation of mRNA Translation by MID1: A Common Mechanism of Expanded CAG Repeat RNAs
Source: Front Cell Neurosci. 2016 Oct 7;10:226. doi: 10.3389/fncel.2016.00226 (PMC5054010; doi:10.3389/fncel.2016.00226)
Supplement: Supplementary Table 1 — ATXN2, ATXN3, and ATXN7-constructs. The sequences of the fragments of ATXN2, ATXN3, and ATXN7 normal and mutant that were cloned into the reporter plasmids and were used in this study are shown. [file Table1.DOCX]

ATXN2 normal

CCTCACCATGTCGCTGAAGCCCCAGCAGCAGCAGCAGCAGCAGCAGCAACAGCAGCAGCAGCAACAGCAGCAGCAGCAGCAGCAGCAGCCGCCGCCCGCGGCTGCCAATGTCCGCAAGCCCGGCGGCAGCGGCCTTCTAGCGTCGCCCGCCGCCGCGCCTTCGCCGTCCTCGTCCTCGGTCTCCTCGTCCTCGGCCACGGCTCCCTCCTCGGTGGTCGCGGCGACCTCCGGCGGCGGGAGGCCCGGCCTGGGCAGAGGTCGAAACAGTAA

ATXN2 mutant

CCTCACCATGTCGCTGAAGCCCCAGCAGCAGCAGCAGCAGCAGCAGCAGCAGCAGCAGCAGCAGCAGCAGCAGCAGCAGCAGCAGCAGCAGCAGCAGCAGCAGCAGCAGCAGCAGCAGCAGCAGCAGCAGCAGCAGCAGCAGCAGCAGCAGCAGCAGCAGCAGCAGCAGCAGCAGCAGCAGCAGCAGCAGCAGCAGCAGCAGCAGCAGCAGCAGCAGCAGCAGCAGCAGCAGCAGCAGCAGCAGCCGCCGCCCGCGGCTGCCAATGTCCGCAAGCCCGGCGGCAGCGGCCTTCTAGCGTCGCCCGCCGCCGCGCCTTCGCCGTCCTCGTCCTCGGTCTCCTCGTCCTCGGCCACGGCTCCCTCCTCGGTGGTCGCGGCGACCTCCGGCGGCGGGAGGCCCGGCCTGGGCAGAGGTCGAAACAGTAA

ATXN3 normal

CCGCAGGGCTATTCAGCTAAGTATGCAAGGTAGTTCCAGAAACATATCTCAAGATATGACACAGACATCAGGTACAAATCTTACTTCAGAAGAGCTTCGGAAGAGACGAGAAGCCTACTTTGAAAAACAGCAGCAAAAGCAGCAGCAGCAGCAGCAGCAGCAGCAGCAGCAGCAGCAGCAGCAGGGGGACCTATCAGGACAGAGTTCACATCCATGTGAAAGGCCAGCCACCAGTTCAGGAGCACTTGGGAGTGATCTAGGTGATGCTATGAGTGAAGAAGACATGCTTCAGGCAGCTG

ATXN3 mutant

CCGCAGGGCTATTCAGCTAAGTATGCAAGGTAGTTCCAGAAACATATCTCAAGATATGACACAGACATCAGGTACAAATCTTACTTCAGAAGAGCTTCGGAAGAGACGAGAAGCCTACTTTGAAAAACAGCAGCAAAAGCAGCAACAGCAGCAGCAGCAGCAGCAGCAGCAGCAGCAGCAGCAGCAGCAGCAGCAGCAGCAGCAGCAGCAGCAGCAGCAGCAGCAGCAGCAGCAGCAGCAGCAGCAGCAGCAGCAGCAGCAGCAGCGGCAGCAGCAGCAGCAGCAGCAGCAGCAGCAGCAGCAGCAGCAGCAGCAGCAGCAGCAGCAGCAGCGGGACCTATCAGGACAGAgTTCACATCCATGTGAAAGGCCAGCCACCAGTTCAGGAGCACTTGGGAGTGATCTAGGTGATGCTATGAGTGAAGAAGACATGCTTCAGGCAGCTG

ATXN7 normal

ATGTCGGAGCGGGCCGCGGATGACGTCAGGGGGGAGCCGCGCCGCGCGGCGGCGGCGGCGGGCGGAGCAGCGGCCGCGGCCGCCCGGCAGCAGCAGCAGCAGCAGCAGCAGCAGCAGCAGCAGCAGCAGCAGCAGCAGCAGCAGCCGCCGCCTCCGCAGCCCCAGCGGCAGCAGCACCCGCCACCGCCGCCACGGCGCACACGGCCGGAGGACGGCGGGCCCGGCGCCGCCTCCACCTCGGCCGCCGCAATGGCGACGGTCGGGGAGCGCAGGCCTCTGCCCAGTCCTGAAGTGATGCTGGG

ATXN7 mutant

ATGTCGGAGCGGGCCGCGGATGACGTCAGGGGGGAGCCGCGCCGCGCGGCGGCGGCGGCGGGCGGAGCAGCGGCCGCGGCCGCCCGGCAGCAGCAGCAGCAGCAGCAGCAGCAGCAGCAGCAGCAGCAGCAGCAGCAGCAGCAGCAGCAGCAGCAGCAGCAGCAGCAGCAGCAGCAGCAGCAGCAGCAGCAGCAGCAGCAGCAGCAGCAGCAGCAGCGGCAGCAGCAGCAGCAGCAGCAGCAGCAGCAGCAGCAGCAGCAGCAGCAGCAGCAGCAGCAGCAGCAGCAGCAGCAGCAGCAGCAGCAGCAGCAGCAGCAGCAGCAGCAGCAGCCGCCGCCTCCGCAGCCCCAGCGGCAGCAGCACCCGCCACCGCCGCCACGGCGCACACGGCCGGAGGACGGCGGGCCCGGCGCCGCCTCCACCTCGGCCGCCGCAATGGCGACGGTCGGGGAGCGCAGGCCTCTGCCCAGTCCTGAAGTGATGCTGGG
